# Longitudinal alterations of the gut mycobiota and microbiota on COVID-19 severity

**DOI:** 10.1186/s12879-022-07358-7

**Published:** 2022-06-24

**Authors:** Yuichi Maeda, Daisuke Motooka, Takahiro Kawasaki, Hiroya Oki, Yoshimi Noda, Yuichi Adachi, Takayuki Niitsu, Shota Okamoto, Kentaro Tanaka, Kiyoharu Fukushima, Saori Amiya, Reina Hara, Eri Oguro-Igashira, Takanori Matsuki, Haruhiko Hirata, Yoshito Takeda, Hiroshi Kida, Atsushi Kumanogoh, Shota Nakamura, Kiyoshi Takeda

**Affiliations:** 1 Laboratory of Immune Regulation, Department of Microbiology and Immunology, Graduate School of Medicine, Osaka University, Suita, Japan; 2 WPI Immunology Frontier Research Center, Osaka University, Suita, Japan; 3 Department of Respiratory Medicine, Clinical Immunology, Graduate School of Medicine, Osaka University, Suita, Japan; 4 Integrated Frontier Research for Medical Science Division, Institute for Open and Transdisciplinary Research Initiatives, Osaka University, Suita, Japan; 5 Department of Infection Metagenomics, Genome Information Research Center, Research Institute for Microbial Diseases, Osaka University, Suita, Japan; 6 National Hospital Organization Osaka Toneyama Medical Center, Osaka, Japan; 7 Department of Host Defense, Research Institute for Microbial Diseases (RIMD), Osaka University, Suita, Japan; 8 Center for Infectious Disease Education and Research, Osaka University, Suita, Japan

**Keywords:** COVID-19, Mycobiota, Microbiota, Gut, *Candida*, *Enterococcus*

## Abstract

**Background:**

The impact of SARS-CoV-2 infection on the gut fungal (mycobiota) and bacterial (microbiota) communities has been elucidated individually. This study analyzed both gut mycobiota and microbiota and their correlation in the COVID-19 patients with severe and mild conditions and follow-up to monitor their alterations after recovery.

**Methods:**

We analyzed the gut mycobiota and microbiota by bacterial 16S and fungal ITS1 metagenomic sequencing of 40 severe patients, 38 mild patients, and 30 healthy individuals and reanalyzed those of 10 patients with severe COVID-19 approximately 6 months after discharge.

**Results:**

The mycobiota of the severe and mild groups showed lower diversity than the healthy group, and in some, characteristic patterns dominated by a single fungal species, *Candida albicans*, were detected. Lower microbial diversity in the severe group was observed, but no differences in its diversity or community structure were detected between the mild and healthy groups. The microbiota of the severe group was characterized by an increase in *Enterococcus* and *Lactobacillus*, and a decrease in *Faecalibacterium* and *Bacteroides*. The abundance of *Candida* was positively correlated with that of *Enterococcus* in patients with COVID-19*.* After the recovery of severe patients, alteration of the microbiota remained, but the mycobiota recovered its diversity comparable to that of mild and healthy groups.

**Conclusion:**

In mild cases, the microbiota is stable during SARS-CoV-2 infection, but in severe cases, alterations persist for 6 months after recovery.

**Supplementary Information:**

The online version contains supplementary material available at 10.1186/s12879-022-07358-7.

## Introduction

Coronavirus disease 2019 (COVID-19) is caused by severe acute respiratory syndrome coronavirus 2 (SARS-CoV-2). More than 100 million people worldwide have been infected by SARS-CoV-2 [1]. It has been reported that dysregulated immune responses correlate with the severity of COVID-19 [2].

The gut fungal community (mycobiota) plays an important role in host immune responses and the prevention of infection [3, 4]. An altered mycobiota composition has been observed in patients with COVID-19 [5, 6]. Patients with severe COVID-19 were usually treated with dexamethasone, which may affect the gut mycobiota [7]. *Candida* and *Aspergillus* species were enriched in fecal samples from hospitalized patients with COVID-19 [5]. Secondary infections, such as COVID-19-associated candidemia, present serious complications in the treatment of severe disease [8]. Previous reports found that the mortality rate is high in patients with COVID-19-associated candidemia [9, 10]. However, it is still unclear how disease severity affects the mycobiota composition.

The gut bacterial community (microbiota) also plays an important role in regulating innate and adaptive immune responses during infection [11, 12]. Several studies reported alteration of the gut microbiota composition in patients with COVID-19 [13–17]. These changes were characterized by the increase of opportunistic bacteria and the decrease of beneficial commensal bacteria. Some of these changes were possible due to the use of antibiotics [16]. Some specific bacteria were correlated with disease severity [14]. Thus, gut microbiota alterations might reflect the clinical outcome of COVID-19. However, no study has investigated the correlation between mycobiota and microbiota, and the severity of COVID-19.

Alteration of the gut microbiota in response to respiratory infections by other viruses has also been reported. For example, patients with influenza A virus infection displayed lower diversity and a different composition of the intestinal microbiota than healthy controls [13]. It remains unclear whether the altered composition of the mycobiota and microbiota in patients with COVID-19 is indicative of the sensitivity to SARS-CoV-2 infection or simply a consequence of the inflammatory condition of viral infection.

There is limited evidence of the altered microbiota composition in patients who recovered from COVID-19. A report demonstrated that the diversity and community structure of the gut microbiota in patients with COVID-19 at 3 months after discharge was different from that of healthy controls [18]. Another study showed prolonged impairment of fecal metabolites and microbiota after a month of the discharge in patients with COVID-19 [19]. To understand the long lasting COVID-19 symptoms, a longitudinal study of the gut mycobiota and microbiota in patients with severe COVID-19 is required.

This study explored the gut mycobiota and microbiota in patients who recovered from COVID-19. We first investigated the correlation between gut mycobiota and microbiota in hospitalized patients with severe and mild conditions. In addition, we collected fecal samples from 10 patients who had the severe disease after approximately 6 months of recovery and reanalyzed their mycobiota and microbiota alterations.

## Materials and methods

### Study participants

Fecal samples were obtained from 40 patients with severe COVID-19 at Osaka University Hospital and 38 patients with mild COVID-19 at Osaka Toneyama Medical Center. The severity of COVID-19 was categorized as (1) mild, if there was no radiographic evidence of pneumonia; (2) moderate, if pneumonia was present without requiring mechanical ventilation or intensive care; (3) severe, if there was respiratory failure requiring mechanical ventilation, shock, or organ failure requiring intensive care. In this study, “mild” included patients with mild-to-moderate COVID-19. Fecal samples were recollected from 10 patients with severe COVID-19 after discharge. Fecal samples were also collected from 30 age- and sex-matched healthy controls. Samples were collected after informed consent was obtained from the subjects in accordance with the Declaration of Helsinki, and the study was conducted with approval from the local ethics committees of Osaka University Hospital and Osaka Toneyama Medical Center.

### Extraction of bacterial and fungal DNA

Human fecal samples were collected in sterile transparent tubes with a screw cap (L × Ø: 76 × 20 mm) (SARSTEDT) containing RNA*later*® (Ambion). After samples were weighed, RNA*later*® was added to generate tenfold dilutions of homogenates. Homogenates (200 µl) were washed twice with 1 ml of phosphate-buffered saline and stored at − 20 °C until use. Bacterial DNA was extracted according to a previously described method [20]. Briefly, to extract DNA, 300 µl of Tris-SDS solution, 0.3 g of glass beads (diameter, 0.1 mm, BioSpec Products), and 500 µl of Tris–EDTA-saturated phenol were added to the suspension, and the mixture was vortexed vigorously using a FastPrep-24 (M.P. Biomedicals) at a power level of 5.0 for 30 s for bacterial DNA and 60 s for fungal DNA. After centrifugation at 20,000×*g* for 5 min, the supernatant (400 µl) was collected. Subsequently, phenol–chloroform extraction was performed, and the supernatant of 250 µl was subjected to isopropanol precipitation. Finally, DNA was suspended in 200 µl of TE buffer and stored at − 20 °C.

### qRT-PCR and whole-genome sequencing of SARS2-CoV-2

RNA was extracted from the nasal swab, sputum, or stool samples stored in RNA*later* using a QIAamp Viral RNA Mini Kit (QIAGEN, Hilden, Germany) according to the manufacturer’s instructions. The extracted RNA was then subjected to qRT-PCR targeting the N gene to obtain Ct values. Each reaction mixture consisted of One Step PrimeScript III RT-qPCR Mix (Takara Bio, Shiga, Japan), a forward primer (NIID_2019-nCOV_N_F2, 500 nM), a reverse primer (NIID_2019-nCOV_N_R2, 700 nM), a probe (NIID_2019-nCOV_N_P2, 200 nM), and 2 µl of RNA solution in a final volume of 5 µl.

SARS-CoV-2 positive control RNA for One-Step RT-PCR (#JP-NN2-PC, NIHON GENE RESEARCH LABORATORIES, Sendai, Japan) was used for the standard control of qPCR. An Eco 48 Real-Time qPCR system (PCR max, Stone, Staffordshire, UK) was used for the qRT-PCR assays with the following program: 5 min at 52 °C, 10 s at 95 °C, and 45 cycles of 15 s at 95 °C and 60 s at 60 °C. To determine the SARS-CoV-2 genome sequences, we prepared sequencing libraries using the multiplex PCR method. Briefly, after reverse transcription using SuperScript IV Reverse Transcriptase (Thermo Fisher Scientific, Waltham, MA, USA) and random primers pd(N)6 (Takara Bio), whole-genome amplification was performed using ATRIC Network’s modified (V3) primer set [21]. NGS libraries were prepared using the Nextera XT Library Prep Kit (Illumina, San Diego, CA, USA). Paired-end sequencing to a length of 2 × 150 bp was performed on a DNBSEQ-G400RS sequencer (MGI, Yantian, Shenzhen, China) using the DNBSEQ-G400RS High-throughput Sequencing Kit (FCL PE150). After trimming the adapter sequences using Cutadapt version 3.2, the trimmed sequence reads were aligned to the reference genome of SARS-CoV-2 (GenBank accession number: NC_045512.2) by BWA version 0.7.17. After marking duplicate reads in BAM files using Samtools version 1.11 and Picard in GATK 4.2.0.0, variant calling was executed using Mutect2 in GATK 4.2.0.0. Consensus sequences were obtained using bcftools version 1.9.

### Determination of the bacterial and fungal composition by amplicon deep sequencing

Amplicon libraries were prepared using the two-step tailed PCR method for microbiota analysis by targeting the V1–V2 region of the 16S rRNA gene (27Fmod, 5ʹAGRGTTTGATYMTGGCTCAG-3ʹ; 338R, 5ʹ-TGCTGCCTCCCGTAGGAGT-3ʹ) and for mycobiota analysis by targeting the fungal internal transcribed region 1 (ITS1) region (ITS1-F, 5′-CTTGGTCATTTAGAGGAAGTAA-3′; ITS2, 5′-GCTGCGTTCTTCATCGATGC-3′). Then, 301-bp paired-end sequencing of these amplicons was performed on a MiSeq system (Illumina, San Diego, CA) using a MiSeq Reagent v3 600 cycle kit. The paired-end sequences obtained were merged, filtered, and denoised using DADA2. Taxonomic assignment was performed using QIIME2 feature-classifier plugin with the Greengenes 13_8 database for bacteria and the ntF-ITS1 database for fungi [22]. The QIIME2 pipeline, version 2020.2 was used as the bioinformatics environment to process all relevant raw sequencing data (https://qiime2.org).

### Statistical analysis

Principal coordinate analysis was performed using the R package ade4, and ANOSIM was performed using the *R* package Vegan. The differential in bacterial and fungal taxonomy between groups was identified by linear discriminant analysis effect size (LEfSe) [23].

### Patient and public involvement

Patients or the public were not involved in the design, conduct, reporting, or dissemination plans of this study.

## Results

### Characteristics of patients with severe and mild COVID-19 compared with healthy controls

Clinical characteristics such as comorbidities and symptoms at enrollment are presented in Table 1. Five fatal cases were included in the severe group, whereas no fatal cases were observed in the mild group. Antibiotics were used by 90% of patients with severe disease and 84.2% of patients with mild disease. Dexamethasone was used by 95% of patients with severe disease and 47.4% of patients with mild COVID-19. No patients with mild COVID-19 were treated with antifungal drugs. The clinical information for the severe and mild groups, including the duration of hospitalization, day of stool sampling, and drug usage, is presented in Additional file 1: Figs. S1 and S2. We performed SARS-CoV-2 qRT-PCR on the collected stool specimens, and the results demonstrated that 17 patients in the severe group, but none in the mild group, were positive for coronavirus. Among the positive samples, 12 were evaluated by whole-genome sequencing. The obtained genome sequences illustrated that all SARS-CoV-2 variants were assigned to clade 20B on the Nextclade lineage (Table 2).

**Table 1:** Baseline characteristics and clinical course

	Severe COVID-19	Mild COVID-19	Healthy controls	P value
Numbers of samples	40	38	30	
Median age (IQR), year	72 (62.75–74)	72 (58–78)	64 (61–69.75)	0.21
Female sex-no./total no.	10 (25%)	16 (42.1%)	10 (33.3%)	0.27
Median BMI	23.2	23.6	N.A	0.39
Comorbidities				
Hypertension	19 (47.5%)	13 (34.2%)	8	0.18
Diabetes mellitus	11(27.5%)	6 (15.8%)	4	0.26
Dyslipidemia	11 (27.5%)	12 (31.6%)	6	0.55
Symptoms at admission				
Fever	33 (82.5%)	29 (76.3%)	N.A	0.49
Diarrhea	8 (20%)	4 (10.5%)	N.A	0.34
Respiratory symptoms	10 (25%)	18 (47.4%)	N.A	0.06
Antibiotics therapy	36 (90%)	32(84.2%)	0	< 0.001
Meropenem	20 (50%)	0	0	N.T
Tazobactam/piperacillin	11 (27.5%)	0	0	N.T
Azithromycin	8 (20%)	31 (81.6%)	0	N.T
Levofloxacin	13 (32.5%)	3 (7.9%)	0	N.T
Other antibiotics	14 (35%)	7 (18.4%)	0	N.T
Dexamethasone therapy	38 (95%)	18(47.4%)	0	< 0.001
Median dose (mg)	6	6	0	1
Death	5 (12.5%)	0	N.A	0.05
Sampling days after onset of symptoms, Median (IQR)	13 (9.75–18)	17 (11–22.5)	N.A	0.58

Values are expressed in number (percentage)

*N.A* not applicable, *N.T* not tested

**Table 2:** Genome sequence of SARS-CoV2

Patient ID	Sample type	Ct value	NextClade lineage	Spike amino acid changes
Cov1	Stool	32.9	20B	D614G
Cov2	Stool	34.3	20B	M153T, D614G
Cov3	Stool	40.2	20B	D614G
Cov6	Stool	35.4	20B	D614G
Cov7	Stool	37.0	20B	D614G
Cov9	Stool	36.0	20B	D614G
Cov13	Stool	37.2	20B	R273M, D614G
Cov17	Stool	35.7	20B	R273M, D614G
	Swab	30.8	20B	D614G
	Sputum	25.7	20B	D614G
Cov27	Swab	23.6	20B	D614G
	Sputum	29.9	20B	D614G
Cov32	Stool	30.8	20B	D614G, Q675H
	Swab	16.3	20B	D614G, Q675H
	Sputum	26.7	20B	D614G, Q675H
Cov38	Stool	37.8	20B	D614G
	Swab	18.0	20B	D614G
	Sputum	29.5	20B	D614G
Cov40	Stool	38.0	20B	D614G
	Swab	22.3	20B	D614G
	Sputum	32.3	20B	D614G

### Alteration of the mycobiota in patients with COVID-19

**Fig. 1:**
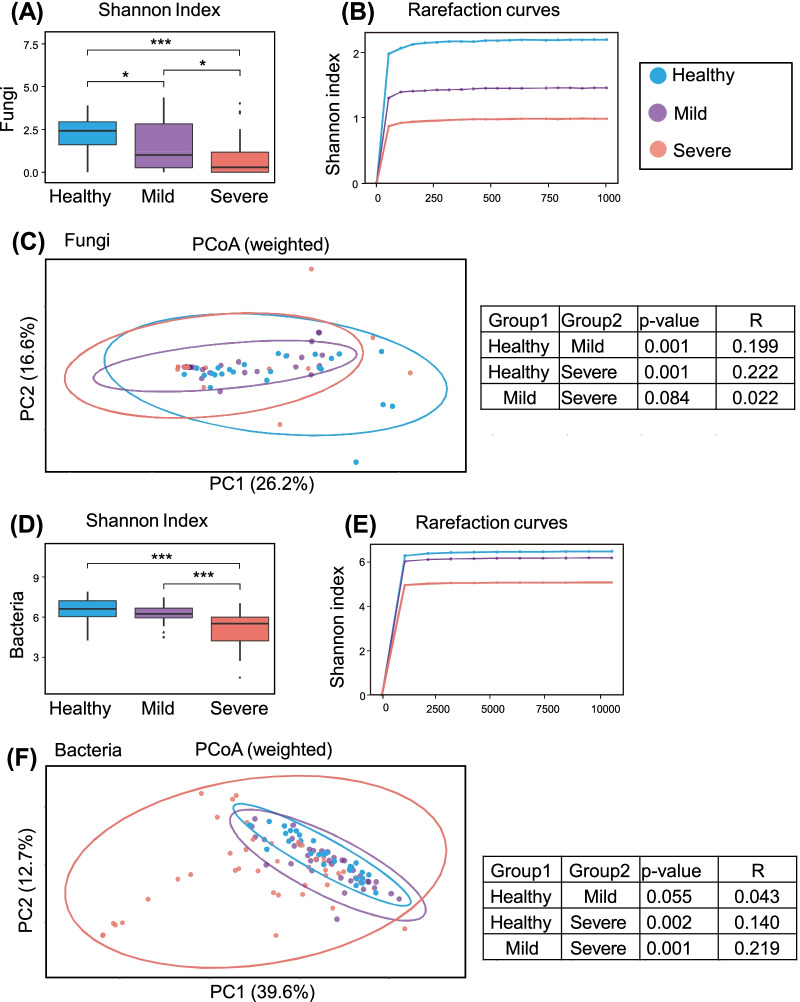
Alterations in gut fungal and bacterial diversity in patients with COVID-19 compared with healthy controls. Diversity of the gut mycobiota (**A**–**C**) among patients with severe (n = 35), mild (n = 31) coronavirus disease 2019 (COVID-19) and healthy controls (n = 24). Diversity of microbiota (**D**–**F**) among patients with severe (n = 40), mild (n = 38) COVID-19 and healthy controls (n = 30). **A**, **D** Shannon index. **B**, **E** Rarefaction curves. **C**, **F** Principal coordinate analysis at amplicon sequence variants (ASV) levels. *P < 0.05, ***P < 0.001

**Fig. 2:**
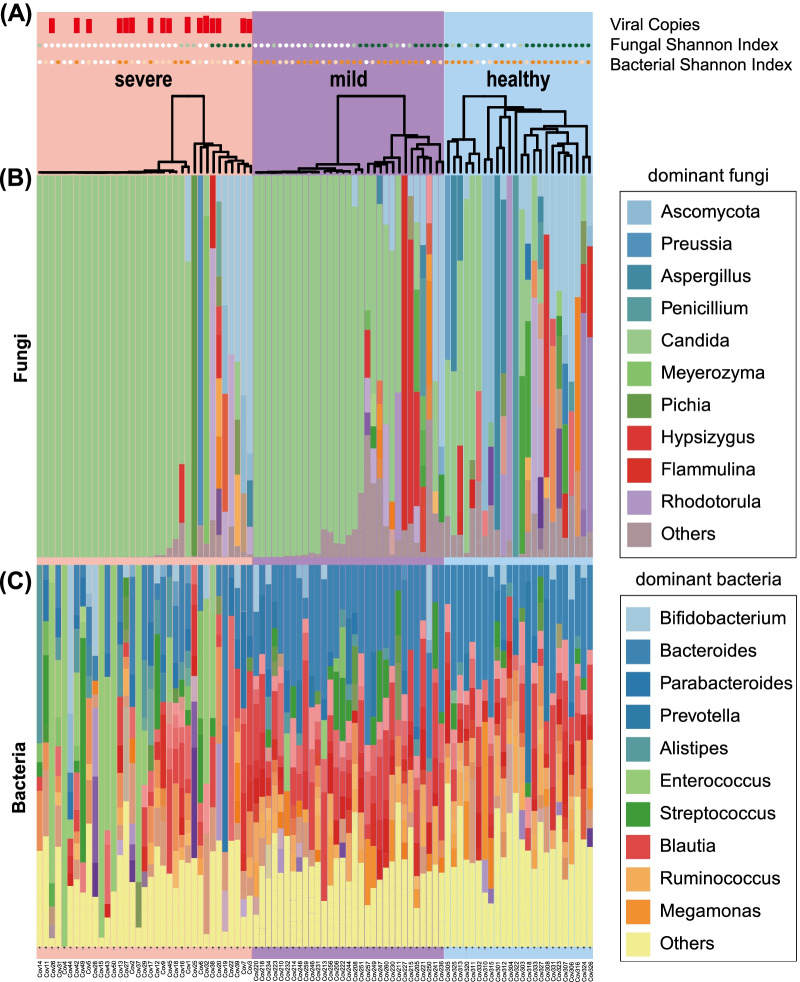
Altered composition of the gut mycobiota and microbiota in patients with COVID-19. **A** The log scale of viral copy number for each subject is illustrated by a red bar chart. Fungal and bacterial Shannon indices are presented as circles with three levels of colors (for fungal Shannon index: < 1, white; 1–2, light green; > 2, dark green; for the bacterial Shannon index: < 5, white; 5–6, light orange, > 6, orange) **B** The composition of major gut mycobiota in patients with severe or mild COVID-19 and healthy controls. **C** The composition of major gut microbiota in patients with severe, or mild COVID-19 and healthy controls

**Fig. 3:**
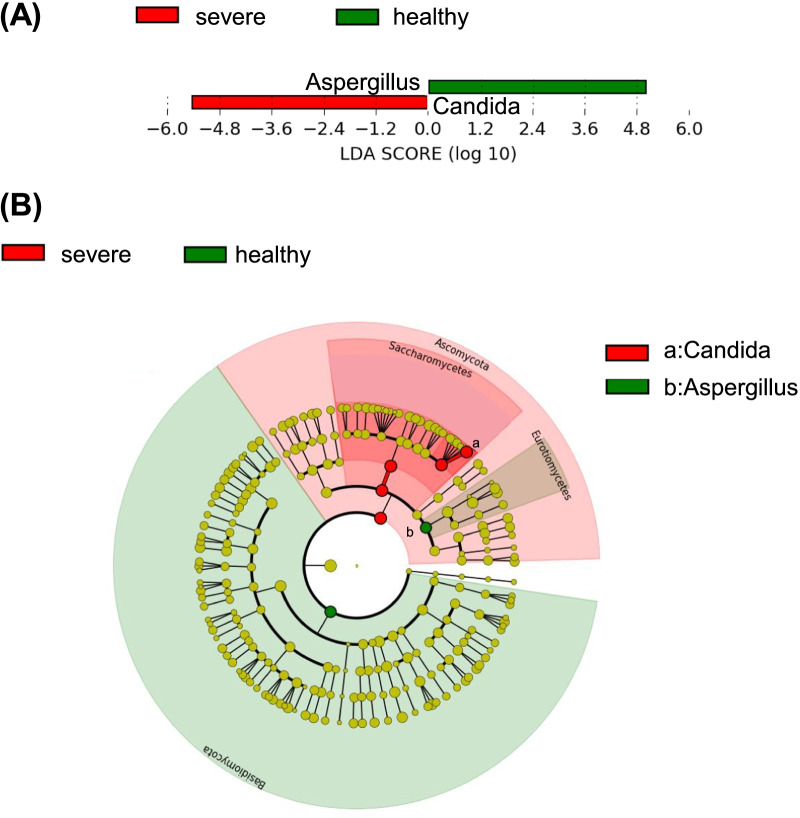
Identifying fungal differences by linear discriminant analysis (LDA) effect size. **A** The LDA score of differentially enriched taxa in patients with severe coronavirus disease 2019 (COVID-19) and healthy controls (HC). **B** Taxonomic cladogram presents the taxa that were considered statistically significant between patients with severe COVID-19 and healthy controls (HC)

We compared the gut mycobiota of patients with severe or mild COVID-19 with that of healthy controls. The fungal α-diversity of the severe disease group was significantly lower than that of the control and mild disease groups (Figs. 1A, B, 2A). The β-diversity of the gut mycobiota in both the severe and mild groups differed from that of the healthy controls (P = 0.001, P = 0.002, respectively; Fig. 1C). The mycobiota was dominated by the genus *Candida* in the severe (60%, 21/35) and mild (51.6%, 16/31) groups, whereas no patients (0/21) in the control group showed a predominance of this genus (Fig. 2B, Additional file 1: Fig. S3). LEfSe identified characteristic fungi in the severe group as *Candida*, whereas *Aspergillus* was identified in the healthy controls (Fig. 3A, B). When analyzing *Candida* species, the patient groups were characterized by an increase in the abundance of *C. albicans* (Additional file 1: Fig. S4). The abundance of other *Candida* species, namely *C. tropicalis*, *C. parapsilosis*, and *C. dubliniensis*, did not significantly differ among the three groups.

To evaluate the effect of dexamethasone, we compared the relative abundance of *Candida* species according to the use of dexamethasone. We observed no significant difference according to the receipt of this drug (Additional file 1: Fig. S5). In addition, 11 patients in the severe group required additional antifungal drugs such as micafungin sodium or amphotericin B, and three patients (Cov-6, Cov-42, and Cov-50) developed candidemia during hospitalization. Ten patients in the severe group received antifungal drugs after the collection of fecal samples. These results indicated that the increased abundance of *Candida* species in patients with COVID-19 was not simply attributable to drug treatment. We also investigated whether antibiotics affect the gut mycobiota in the patients with severe COVID-19. To this end, we divided the patients with severe disease into two groups with or without meropenem, the most frequently used antibiotics. We found that the α- and β-diversity of the gut mycobiota did not differ between the two groups (Additional file 1: Fig. S6A, B).

### Alteration of the fecal microbiota in patients with COVID-19

**Fig. 4:**
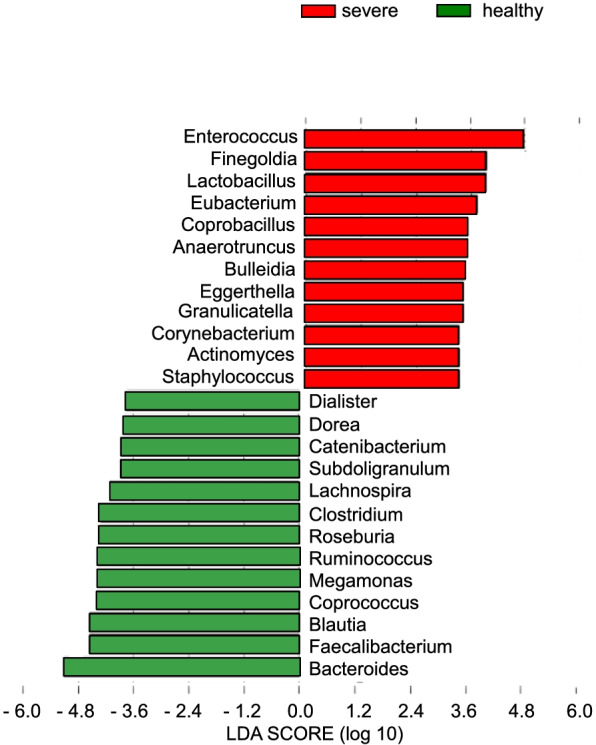
Identifying bacterial differences by linear discriminant analysis (LDA) effect size. The LDA score of differentially enriched taxa in patients with severe coronavirus disease 2019 (COVID-19) patients and healthy controls (HC)

**Fig. 5:**
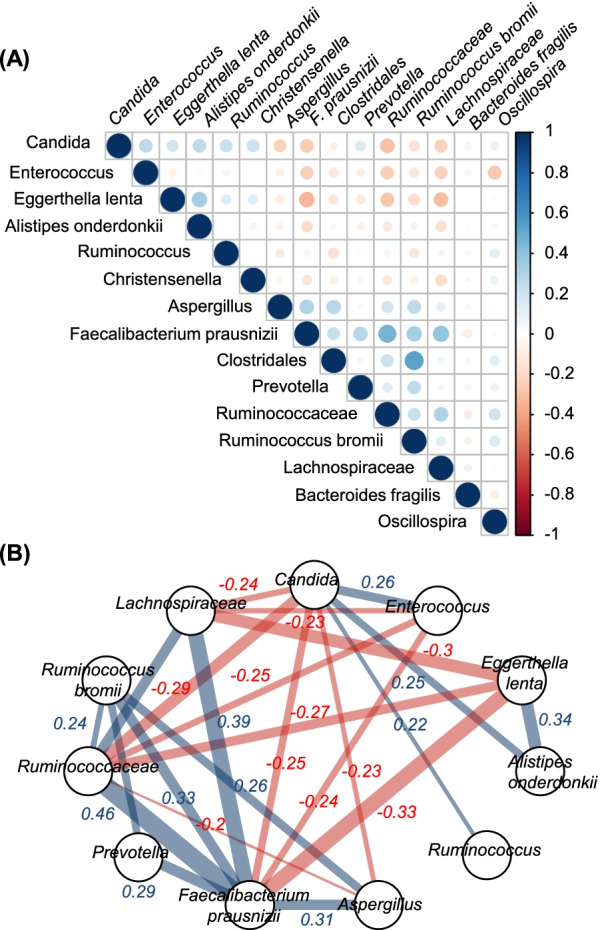
Correlations of gut fungi and bacteria in patients with coronavirus disease 2019 (COVID-19). **A** Correlation plot for associations between two fungal genera (*Candida* and *Aspergillus*) and bacteria. **B** Pairwise correlation network between two fungal genera (*Candida* and *Aspergillus*) and bacteria. Bacteria that were present in 5% or more of the samples were extracted. Correlation coefficients greater than 0.2 or smaller than -0.2 are presented

To evaluate the interaction between fungi and bacteria in patients with COVID-19, we analyzed the gut microbiota of those three groups. The bacterial α-diversity was significantly lower in the severe group than in the control and mild groups (Figs. 1D, E, 2A). The bacterial β-diversity in the severe group differed from those of the mild and control groups (Fig. 1F). There were no significant differences in α- and β-diversity between the mild and control groups. In the severe group, a loss of diversity was observed for individual genera, such as *Enterococcus*, *Alistipes*, and *Streptococcus*, accounting for over 50% of the microbiota (Fig. 2C, Additional file 1: Fig. S7). No such patterns were observed in the mild and healthy groups. LEfSe identified characteristic bacteria in the severe group as *Enterococcus*, *Finegoldia*, and *Lactobacillus*. The characteristic bacteria in the healthy controls were identified as *Bacteroides*, *Faecalibacterium*, and *Blautia* (Fig. 4). We also investigated whether antibiotics affect the gut microbiota in the patients with severe COVID-19. We found that the use of meropenem did not alter α- and β-diversity of the gut microbiota in patients with severe COVID-19 (Additional file 1: Fig. S6C, D). Moreover, the relative abundance of *Enterococcus*, *Lactobacillus*, and *Finegoldia* was not different between the two groups (Additional file 1: Fig. S6E–G). We next analyzed correlations between the microbiota and mycobiota in all groups. The abundance of the fungus *Candida* was positively correlated with that of *Enterococcus*, *Alistipes onderdonkii*, *Ruminococcus*, and *Eggerthella lenta* and negatively correlated with the fungus *Aspergillus* and the bacteria *Faecalibacterium prausnitzii* and *Lachnospiraceae* (Fig. 5A, B). These results demonstrated that in patients with COVID-19, both mycobiota and microbiota changes were positively correlated with the abundance of *Candida* and *Enterococcus*.

### Longitudinal alteration of the gut microbiota and mycobiota in patients who recovered from COVID-19

**Fig. 6:**
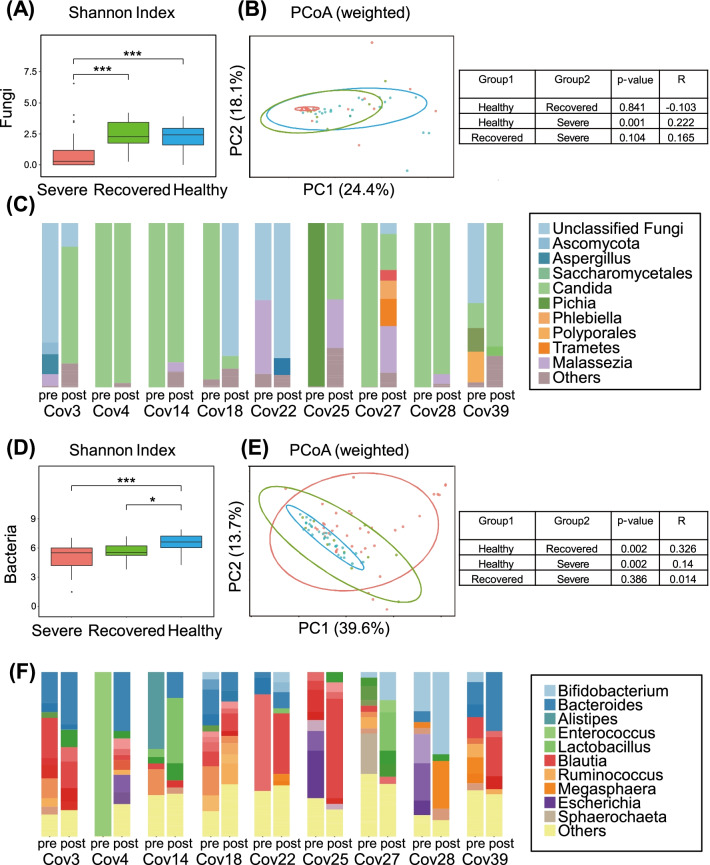
Longitudinal alterations of the gut mycobiota and microbiota in patients with COVID-19. Diversity of the gut mycobiota (**A**, **B**) among patients with severe COVID-19 (severe; n = 35), patients who recovered from COVID-19 (recovered; n = 10) COVID-19, and healthy controls (healthy; n = 24). Diversity of the gut microbiota (**D**, **E**) among patients with severe COVID-19 (severe; n = 40), patients who recovered from COVID-19 (recovered; n = 10) COVID-19, and healthy controls (healthy; n = 30). **C** Fungal composition in patients with severe COIVD-19 at baseline (pre) and after 6 months of recovery (post). **F** The bacterial composition of patients with severe COVID-19 at baseline (pre) and after 6 months of recovery (post)

To investigate whether the altered composition of the mycobiota and microbiota is sustained after recovery from severe COVID-19, we collected fecal samples from 10 patients in the severe group approximately 6 months after recovery. Table 3 presents the characteristics of these patients. We first analyzed the composition of fungi in the recovered patients. The fungal α-diversity of the recovered group was comparable to that of the control group (Fig. 6A). The fungal β-diversity of the recovered group displayed no apparent difference from that in the severe and control groups (Fig. 6B). Dominance by the fungus *Candida* was observed in 56% (5/9) of patients in the recovered group (Fig. 6C). Although the abundance of *Aspergillus* was slightly higher in the recovered group, there was no significant difference between the recovered and control groups (Additional file 1: Fig. S8A). In summary, the α- and β-diversity of the mycobiota in the recovered group reached control levels; however, the increased abundance of *Candida* species was sustained after recovery.

**Table 3:** Clinical characteristics of recovered COVID-19 patients

Patient ID	Age	Gender	Duration from 1st to 2nd sampling (days)	Comorbidities
Cov3	58	Male	241	Hypertension, hyperuricemia, fatty liver
Cov4	68	Male	244	None
Cov10	74	Male	222	Hypertension, dyslipidemia, hyperuricemia
Cov14	73	Male	71	Hypertension, DM
Cov18	64	Male	153	Dilated cardiomyopathy
Cov22	76	Male	144	RA (no treatment), DM
Cov25	56	Male	180	Hypertension, dyslipidemia
Cov27	72	Female	155	Dyslipidemia
Cov28	63	Female	155	DM, dyslipidemia, hashimoto-disease
Cov39	50	Male	120	None

*RA* rheumatoid arthritis, *DM* diabetes mellitus type 2

The bacterial α-diversity of the recovered group increased slightly; however, it remained different from that of the control group (Fig. 6D). The bacterial β-diversity of the recovered group differed significantly from that of the control (P = 0.002) but not from that of the severe disease group (Fig. 6E). The relative abundance of *Lactobacillus* was dominant in some recovered patients (Fig. 6F). The bacteria *Enterococcus* and *A. onderdonkii*, which exhibited higher abundance in the severe group, had a lower abundance in the recovered group (Additional file 1: Fig. S8B). The relative abundance of *Lachnospiraceae* and *F. prausnitzii* in the recovered group was lower than that of the control group. These results indicate that the impact of COVID-19 infection on the microbiota persists after 6 months of recovery.

## Discussion

In the present study, we analyzed both the mycobiota and microbiota of three groups, patients with severe and mild COVID-19 and healthy individuals, in Japan. The mycobiota of patients with severe and mild COVID-19 showed lower diversity, in some cases with *Candida* species, especially *C. albicans*, dominating the composition. Cohorts from Hong Kong and Hangzhou, China, have also reported altered mycobiota composition of COVID-19 patients. The Hangzhou cohort showed lower mycobial diversity and enrichment of *C. albicans*, *C. auris*, and *A. flavus* in COVID-19 patients [6]. In contrast to this study and our study, the Hong Kong cohort demonstrated higher mycobial diversity and enrichment of *C. glabrata* in COVID-19 patients, whereas *Penicillium* and *Aspergillus* were reduced in abundance. The common observation from these studies is the relatively higher composition of *Candida* species in COVID-19 patients.

Patients in the severe group exhibited a high abundance of opportunistic bacteria such as *Enterococcus* and *Lactobacillus* in our cohort. *Enterococcus* causes nosocomial infections, and is a biomarker for poor survival in patients after allogeneic hematopoietic stem cell transplantation [30, 31]. Another report also illustrated that the abundance of opportunistic pathogens such as *Streptococcus*, *Rothia*, and *Veillonella* was elevated in patients with COVID-19 [13]. We found that the abundance of *Candida* was positively correlated with that of *Enterococcus* in patients with COVID-19. It has been reported that in immunocompromised mice, inoculation with *C. albicans* resulted in the overgrowth of *Enterococcus* in the intestine, indicating that these microorganisms synergistically exist in a dysbiotic state [24]. Another report demonstrated that *Enterococcus*-derived products changed the hyphal morphogenesis of *C. albicans*, which was explained by interkingdom signaling [25]. Therefore, it is possible that the abundance of *Candida* and *Enterococcus* coordinately increased in patients with severe COVID-19.

Our study illustrated that alteration in the gut mycobial and microbial compositions persisted for at least 6 months. By then, the mycobial diversity was recovering, but the dominance of *C. albicans* remained. The microbial diversity was also not restored, and the relative abundance of beneficial microbes such as *Faecalibacterium* or *Lachnospiraceae*, was depressed. A possible reason for the sustained alteration of the gut microbiota is inflammation. Indeed, long-lasting COVID-19 symptoms, known as “long COVID,” represent a growing issue. A study found that 13.3% of patients with COVID-19 exhibited symptoms for more than 28 days, and 2.3% of patients experienced symptoms for more than 12 weeks [26]. Thus, the sustained alteration of the gut mycobiota and microbiota might be a cause of long COVID-19 symptoms.

One possible explanation for the altered gut mycobiota is treatment with antifungal drugs. In this study, 3 of 40 patients in the severe group underwent candidemia episodes. Two of three candidemia patients showed a high composition over 97% of intestinal *C. albicans*. The mechanisms for the development of candidemia in some severe COVID-19 patients remain to be elucidated; however immunocompromised condition might be associated with this symptom. Because candidemia is associated with elevated mortality rates in the treatment of COVID-19 [27], 11 patients in the severe group required antifungal drugs. Among them, 10 patients received the antifungal drugs after the sampling. Therefore, the effect of antifungal drugs on the altered mycobiota can be ruled out from our results. The Hangzhou cohort excluded cases in which antifungal drugs were used. The Hong Kong cohort described the use of antibiotics and antiviral drugs, but the usage of antifungal drugs was not mentioned. We show that the mycobiota in the mild group differed from that of the healthy group even though they were not treated with antifungal drugs. In addition, the mycobial compositions dominated by *C. albicans* were observed both in the severe and mild groups. These facts indicate that the use of antifungal drugs is not involved in the altered gut mycobiota.

It has been reported that the use of prednisolone, a type of glucocorticoid, increases *C. albicans* in the murine intestine [28]. We also used a glucocorticoid drug, dexamethasone, for treating most patients in the severe group and approximately half of the patients in the mild group. The Hangzhou cohort also used glucocorticoids in 73.1% of patients. Although it is not explicitly stated, the Hong Kong cohort may not have used immunosuppressive drugs because mild or moderate patients were recruited. We found that there was no difference in the *Candida* species composition with or without the use of dexamethasone. Thus, the immunosuppressive drugs, at least dexamethasone, do not appear to be a major factor in mycobiota alterations.

Another factor associated with the alteration of mycobiota is antibiotic treatment. A study using a mouse model showed that broad-spectrum antibiotic treatment (i.e., clindamycin, and cefoperazone) resulted in the overgrowth of *Candida* species [29]. We used narrow-spectrum antibiotics, such as azithromycin and levofloxacin, for the mild group, and broad-spectrum antibiotics, meropenem, and tazobactam/piperacillin are used for the severe group. We found that the composition of *Candida* species and the fungal β-diversity showed no significant difference between the severe and mild groups. The α-diversity of mycobiota in the severe group was reduced, but no difference was observed between the groups with and without meropenem. Thus, the effect of antibiotic use on mycobiota alterations is not considered to be significant.

The gut microbiota could be more affected by antibiotic use. However, we showed no difference in microbial α- and β-diversities between the mild and healthy groups. Yeoh et al. compared the microbiota of 100 COVID-19 and 78 non-COVID-19 patients and showed no obvious difference in composition. Of these 100 COVID-19 patients (8 severe, 45 moderate, and 47 mild), 34 patients were treated with antibiotics. We found a significant change in the microbiota in 40 patients in the severe group compared with those in the mild and healthy groups. The reduced microbial diversity was observed only in the severe group of our cohort. This difference cannot be explained by the use of meropenem. Gu et al. also reported reduced diversity of the microbiota in COVID-19 patients, including 15 with general symptoms and 15 with severe symptoms. We presume that the disease severity of COVID-19 should be the most relevant factor that influences the microbiota.

In summary, we observed alterations in the mycobiota in patients with mild to severe COVID-19, and also alterations in the microbiota in patients with severe COVID-19. These abnormalities in microbial communities persisted even after recovery. Our findings indicate that the mycobiota would be a more sensitive biomarker than the microbiota for the disease severity of COVID-19. Comparisons between studies are difficult because of the different clinical conditions, including the use of antibiotics and immunosuppressive drugs and the severity of disease; however, the overall conclusion is that COVID-19-induced changes to the mycobiota and microbiota correlate with the severity of the infection. Mycobiota and microbiota in the intestine influence the host immune responses, which might be involved in the long-lasting COVID-19 symptoms. Thus, although a more careful investigation of mycobiota and microbiota would be required, our results suggest that an intervention into intestinal mycobiota and microbiota of patients who recovered from severe COVID-19 will be useful for the improvement of the long-lasting COVID-19 symptoms.

## Supplementary Information

**Additional file 1: Figure S1.** Schematic diagram of stool collection and the duration of hospitalization in patients with severe coronavirus disease 2019. Duration of hospitalization, day of stool sampling, and usage of antibiotics or antifungal drugs are shown. Time from symptom onset until death is also presented. **Figure S2.** Schematic diagram of stool collection and duration of hospitalization in patients with mild coronavirus disease 2019. Duration of hospitalization, day of stool sampling, and usage of antibiotics or antifungal drugs are presented. **Figure S3.** A full list of fungal taxa that correspond to Fig. 2B. **Figure S4.** Relative abundance of *Candida* species, *Candida albicans*, *Candida tropicalis*, *Candida parapsilosis*, *Candida dubliniensis*, and *Aspergillus* among patients with severe (Sev), or mild coronavirus disease 2019 and healthy controls (HC). *Candida* species included *C. albicans*, *C. dubliniensis*, *C. metapsilosis*, *C. parapsilosis*, *C. sojae* and *C. tropicalis*. *P < 0.05, ***P < 0.001, *N.S.* not significant. **Figure S5.** Relative abundance of *Candida* species and *Candida albicans* between patients who were treated (DEX+; n = 48) and without dexamethasone (DEX−; n = 18). *N.S.* not significant. **Figure S6.** Diversity of the gut mycobiota (A, B) between patients with (n = 15) or without (n = 15) usage of meropenem (MEPM) in severe COVID-19. Diversity of the gut microbiota (C, D) between patients with (n = 20) or without (n = 20) usage of MEPM in severe COVID-19. (A, C) Shannon index. (B, D) Principal coordinate analysis at amplicon sequence variants (ASV) levels. Relative abundance of *Enterococcus* (E), *Lactobacillus* (F) and *Finegoldia* (G). *P < 0.05, ***P < 0.001. *N.S.* not significant. **Figure S7.** A full list of bacterial taxa that correspond to Fig. 2A. **Figure S8.** (A) Gut fungal composition among patients with severe (n = 35), coronavirus disease 2019 (COVID-19), patients who recovered from COVID-19 (recovered; n = 10) and healthy controls (n = 24). (B) Gut bacterial composition among the severe (n = 40), recovered COVID-19 patients (n = 10) and healthy controls (n = 30). *P < 0.05, ** P < 0.01, *** P < 0.001.

**Additional file 2: Table S1.** Accession ID of SARS-CoV-2 viral sequences.

## Data Availability

The metagenome sequencing datasets are available in the NCBI/EMBL/DDBJ database, accession numbers DRR352589–DRR352807 in DRA013647. The viral genome sequences of SARS-CoV-2 were deposited to GISAID (https://www.gisaid.org/, Additional file 2: Table S1).
