# Supplementary material for: Longitudinal alterations of the gut mycobiota and microbiota on COVID-19 severity
Source: BMC Infect Dis. 2022 Jun 24;22:572. doi: 10.1186/s12879-022-07358-7 (PMC9233337; doi:10.1186/s12879-022-07358-7)
Supplement: Supplementary file 1 — Additional file 1: Figure S1. Schematic diagram of stool collection and the duration of hospitalization in patients with severe coronavirus disease 2019. Duration of hospitalization, day of stool sampling, and usage of antibiotics or antifungal drugs are shown. Time from symptom onset until death is also presented. Figure S2. Schematic diagram of stool collection and duration of hospitalization in patients with mild coronavirus disease 2019. Duration of hospitalization, day of stool sampling, and usage of antibiotics or antifungal drugs are presented. Figure S3. A full list of fungal taxa that correspond to Fig. 2B. Figure S4. Relative abundance of Candida species, Candida albicans, Candida tropicalis, Candida parapsilosis, Candida dubliniensis, and Aspergillus among patients with severe (Sev), or mild coronavirus disease 2019 and healthy controls (HC). Candida species included C. albicans, C. dubliniensis, C. metapsilosis, C. parapsilosis, C. sojae and C. tropicalis. *P < 0.05, ***P < 0.001, N.S. not significant. Figure S5. Relative abundance of Candida species and Candida albicans between patients who were treated (DEX+; n = 48) and without dexamethasone (DEX−; n = 18). N.S. not significant. Figure S6. Diversity of the gut mycobiota (A, B) between patients with (n = 15) or without (n = 15) usage of meropenem (MEPM) in severe COVID-19. Diversity of the gut microbiota (C, D) between patients with (n = 20) or without (n = 20) usage of MEPM in severe COVID-19. (A, C) Shannon index. (B, D) Principal coordinate analysis at amplicon sequence variants (ASV) levels. Relative abundance of Enterococcus (E), Lactobacillus (F) and Finegoldia (G). *P < 0.05, ***P < 0.001. N.S. not significant. Figure S7. A full list of bacterial taxa that correspond to Fig. 2A. Figure S8. (A) Gut fungal composition among patients with severe (n = 35), coronavirus disease 2019 (COVID-19), patients who recovered from COVID-19 (recovered; n = 10) and healthy controls (n = 24). (B) Gut bacterial co [file 12879_2022_7358_MOESM1_ESM.pdf]

Figure S1

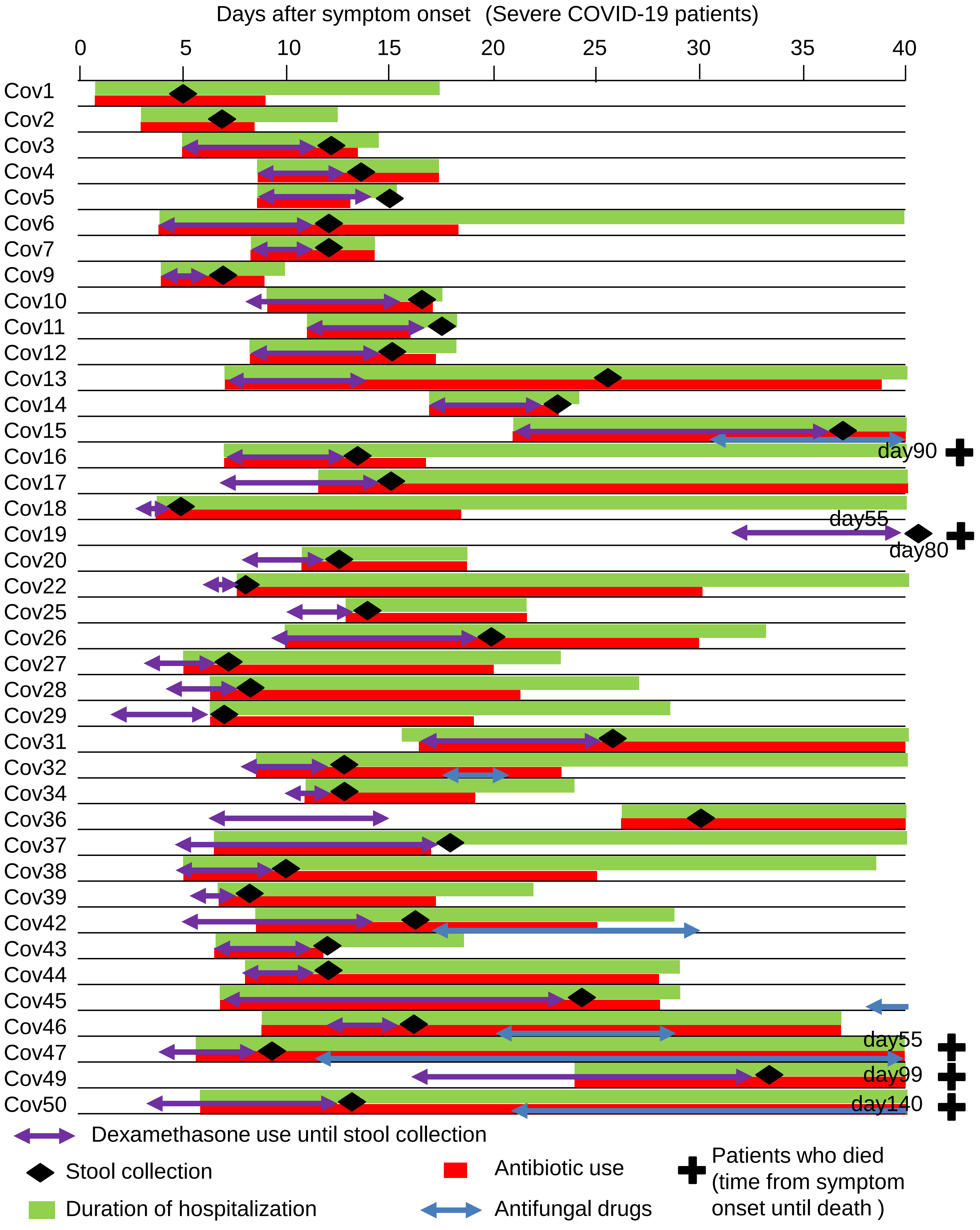
Days after symptom onset (Severe COVID-19 patients)
0
5
10
15
20
25
30
35
40
Cov1
Cov2
Cov3
Cov4
Cov5
Cov6
Cov7
Cov9
Cov10
Cov11
Cov12
Cov13
Cov14
Cov15
Cov16
Cov17
Cov18
Cov19
Cov20
Cov22
Cov25
Cov26
Cov27
Cov28
Cov29
Cov31
Cov32
Cov34
Cov36
Cov37
Cov38
Cov39
Cov42
Cov43
Cov44
Cov45
Cov46
Cov47
Cov49
Cov50
day90
day55
day80
day55
day99
day140
Dexamethasone use until stool collection
Stool collection
Antibiotic use
Patients who died (time from symptom onset until death)
Duration of hospitalization
Antifungal drugs

Figure S2

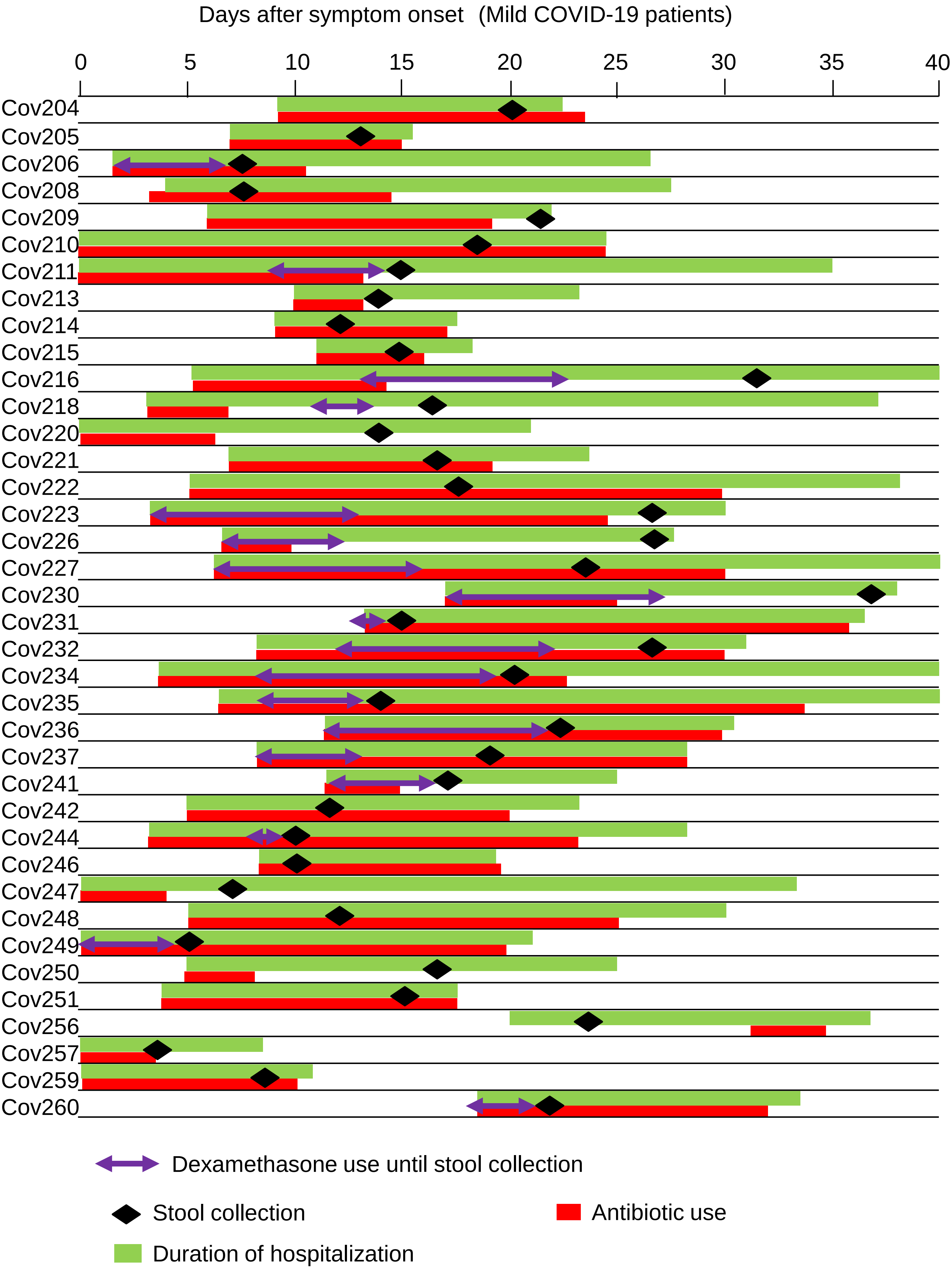

Days after symptom onset (Mild COVID-19 patients)
0
5
10
15
20
25
30
35
40
Cov204
Cov205
Cov206
Cov208
Cov209
Cov210
Cov211
Cov213
Cov214
Cov215
Cov216
Cov218
Cov220
Cov221
Cov222
Cov223
Cov226
Cov227
Cov230
Cov231
Cov232
Cov234
Cov235
Cov236
Cov237
Cov241
Cov242
Cov244
Cov246
Cov247
Cov248
Cov249
Cov250
Cov251
Cov256
Cov257
Cov259
Cov260
Dexamethasone use until stool collection
Stool collection
Antibiotic use
Duration of hospitalization

Figure S3

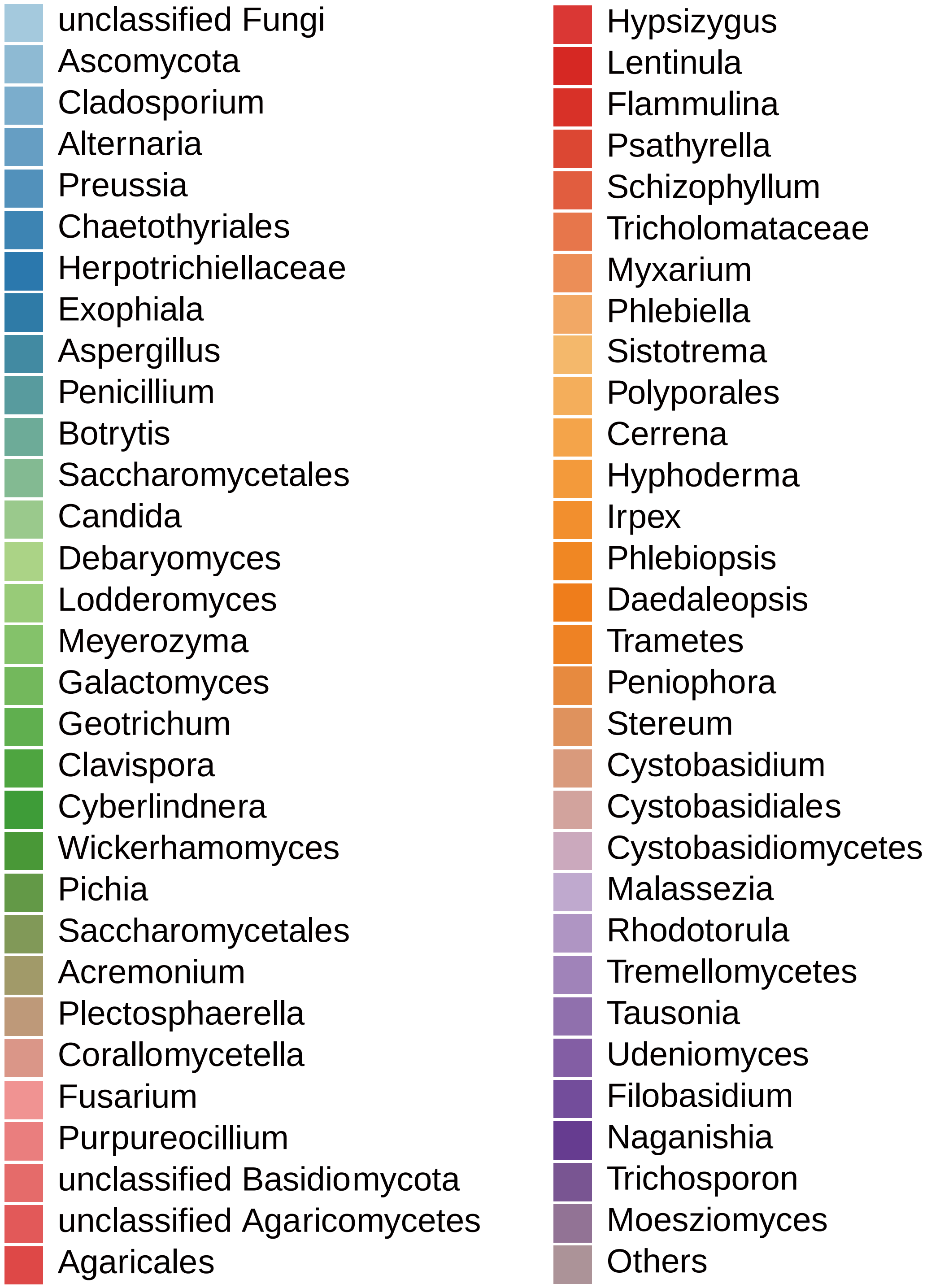
unclassified Fungi
Ascomycota
Cladosporium
Alternaria
Preussia
Chaetothyriales
Herpotrichiellaceae
Exophiala
Aspergillus
Penicillium
Botrytis
Saccharomycetales
Candida
Debaryomyces
Lodderomyces
Meyerozyma
Galactomyces
Geotrichum
Clavispora
Cyberlindnera
Wickerhamomyces
Pichia
Saccharomycetales
Acremonium
Plectosphaerella
Corallomycetella
Fusarium
Purpureocillium
unclassified Basidiomycota
unclassified Agaricomycetes
Agaricales
Hypsizygus
Lentinula
Flammulina
Psathyrella
Schizophyllum
Tricholomataceae
Myxarium
Phlebiella
Sistotrema
Polyporales
Cerrena
Hyphoderma
Irpex
Phlebiopsis
Daedaleopsis
Trametes
Peniophora
Stereum
Cystobasidium
Cystobasidiales
Cystobasidiomycetes
Malassezia
Rhodotorula
Tremellomycetes
Tausonia
Udeniomyces
Filobasidium
Naganishia
Trichosporon
Moesziomyces
Others

Figure S4

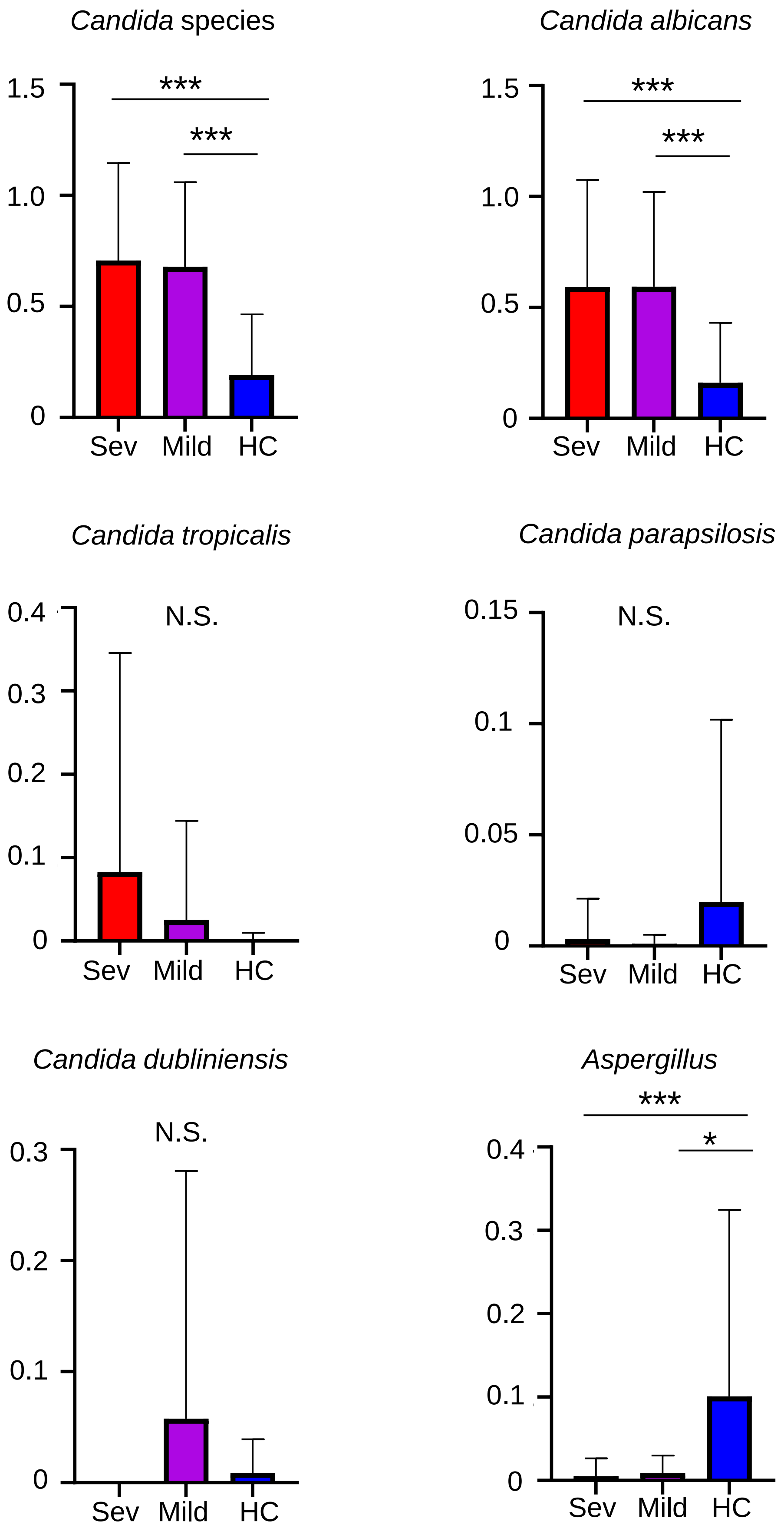

Candida species
Candida albicans
Candida tropicalis
Candida parapsilosis
Candida dubliniensis
Aspergillus
***
*
N.S.
1.5
1.0
0.5
0
0.4
0.3
0.2
0.1
0.15
0.05
Sev
Mild
HC

Figure S5

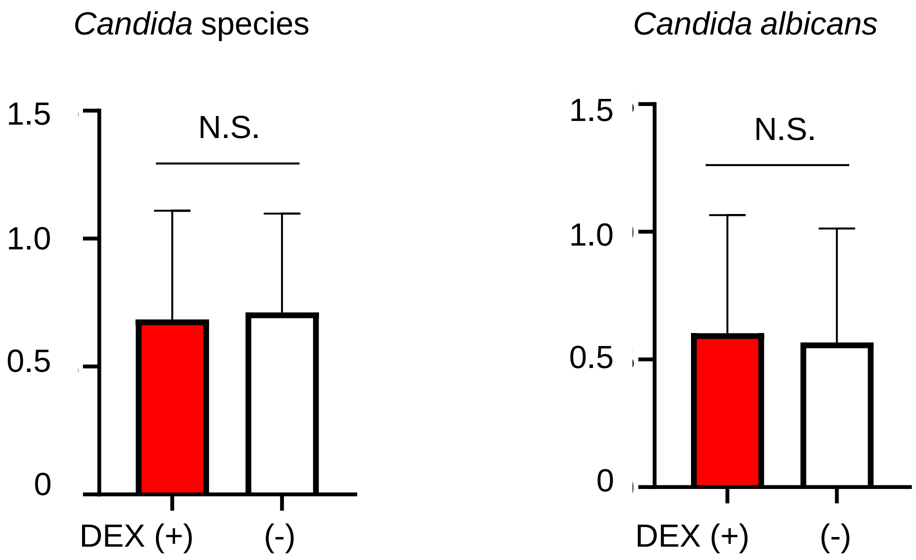
Candida species
1.5
1.0
0.5
0
N.S.
DEX (+)
(-)
Candida albicans
1.5
1.0
0.5
0
N.S.
DEX (+)
(-)

Figure S6

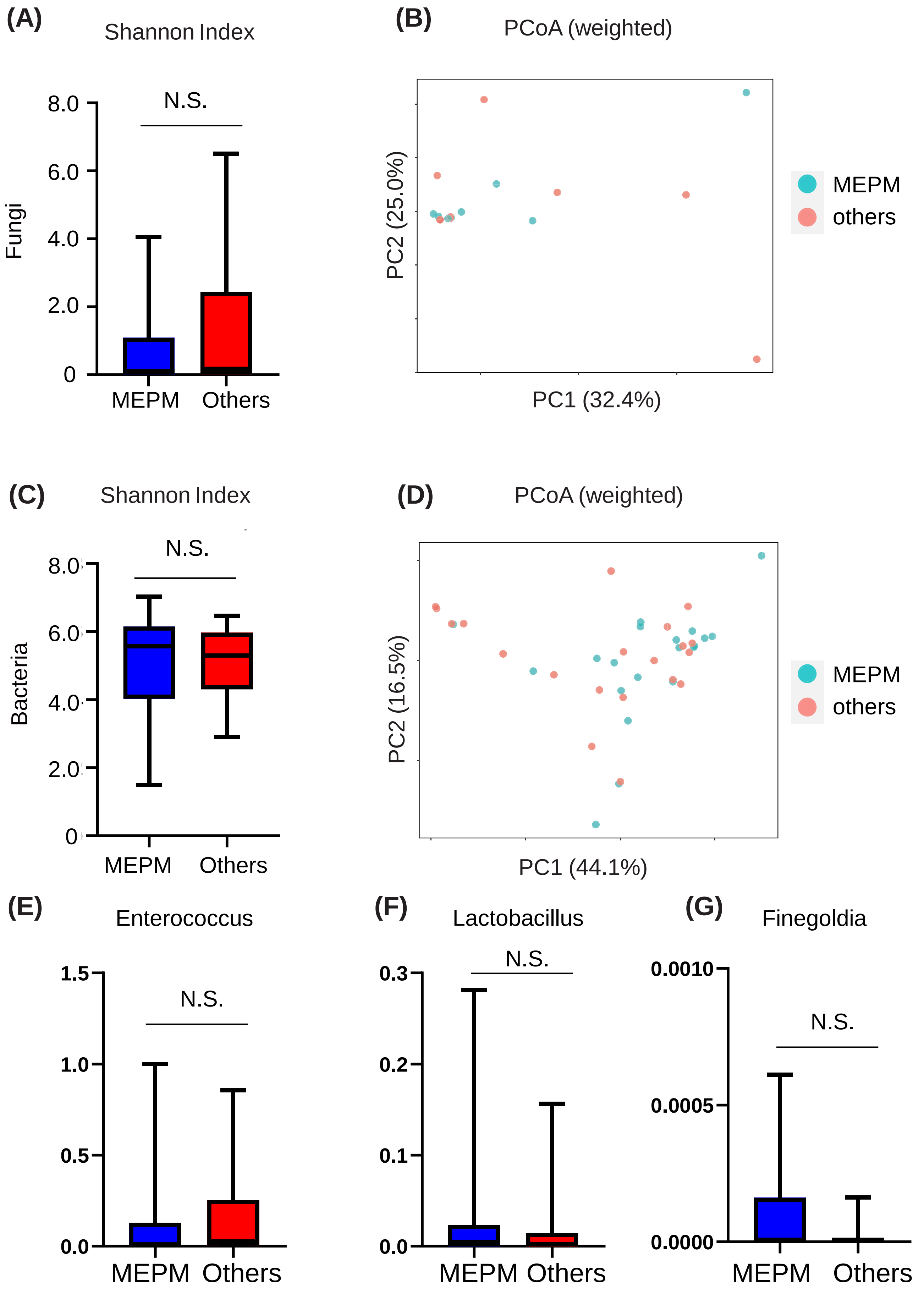
(A)
Shannon Index
N.S.
8.0
6.0
4.0
2.0
0
Fungi
MEPM
Others
(B)
PCoA (weighted)
PC2 (25.0%)
PC1 (32.4%)
MEPM
others
(C)
Shannon Index
N.S.
Bacteria
MEPM
Others
(D)
PCoA (weighted)
PC2 (16.5%)
PC1 (44.1%)
MEPM
others
(E)
Enterococcus
N.S.
1.5
1.0
0.5
0.0
MEPM
Others
(F)
Lactobacillus
N.S.
0.3
0.2
0.1
0.0
MEPM
Others
(G)
Finegoldia
N.S.
0.0010
0.0005
0.0000
MEPM
Others

Figure S7

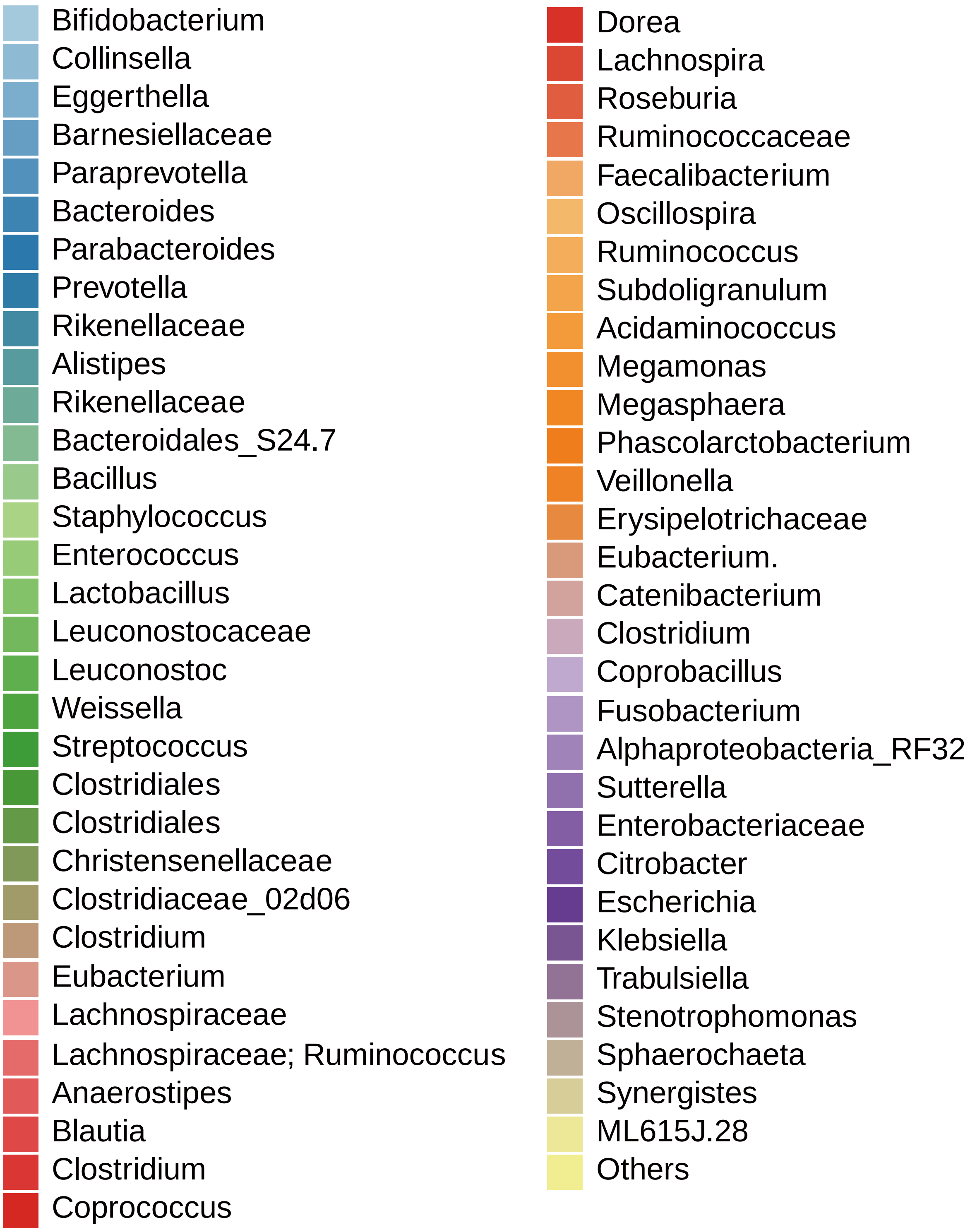
Bifidobacterium
Collinsella
Eggerthella
Barnesiellaceae
Paraprevotella
Bacteroides
Parabacteroides
Prevotella
Rikenellaceae
Alistipes
Rikenellaceae
Bacteroidales_S24.7
Bacillus
Staphylococcus
Enterococcus
Lactobacillus
Leuconostocaceae
Leuconostoc
Weissella
Streptococcus
Clostridiales
Clostridiales
Christensenellaceae
Clostridiaceae_02d06
Clostridium
Eubacterium
Lachnospiraceae
Lachnospiraceae; Ruminococcus
Anaerostipes
Blautia
Clostridium
Coprococcus
Dorea
Lachnospira
Roseburia
Ruminococcaceae
Faecalibacterium
Oscillospira
Ruminococcus
Subdoligranulum
Acidaminococcus
Megamonas
Megasphaera
Phascolarctobacterium
Veillonella
Erysipelotrichaceae
Eubacterium.
Catenibacterium
Clostridium
Coprobacillus
Fusobacterium
Alphaproteobacteria_RF32
Sutterella
Enterobacteriaceae
Citrobacter
Escherichia
Klebsiella
Trabulsiella
Stenotrophomonas
Sphaerochaeta
Synergistes
ML615J.28
Others

Figure S8

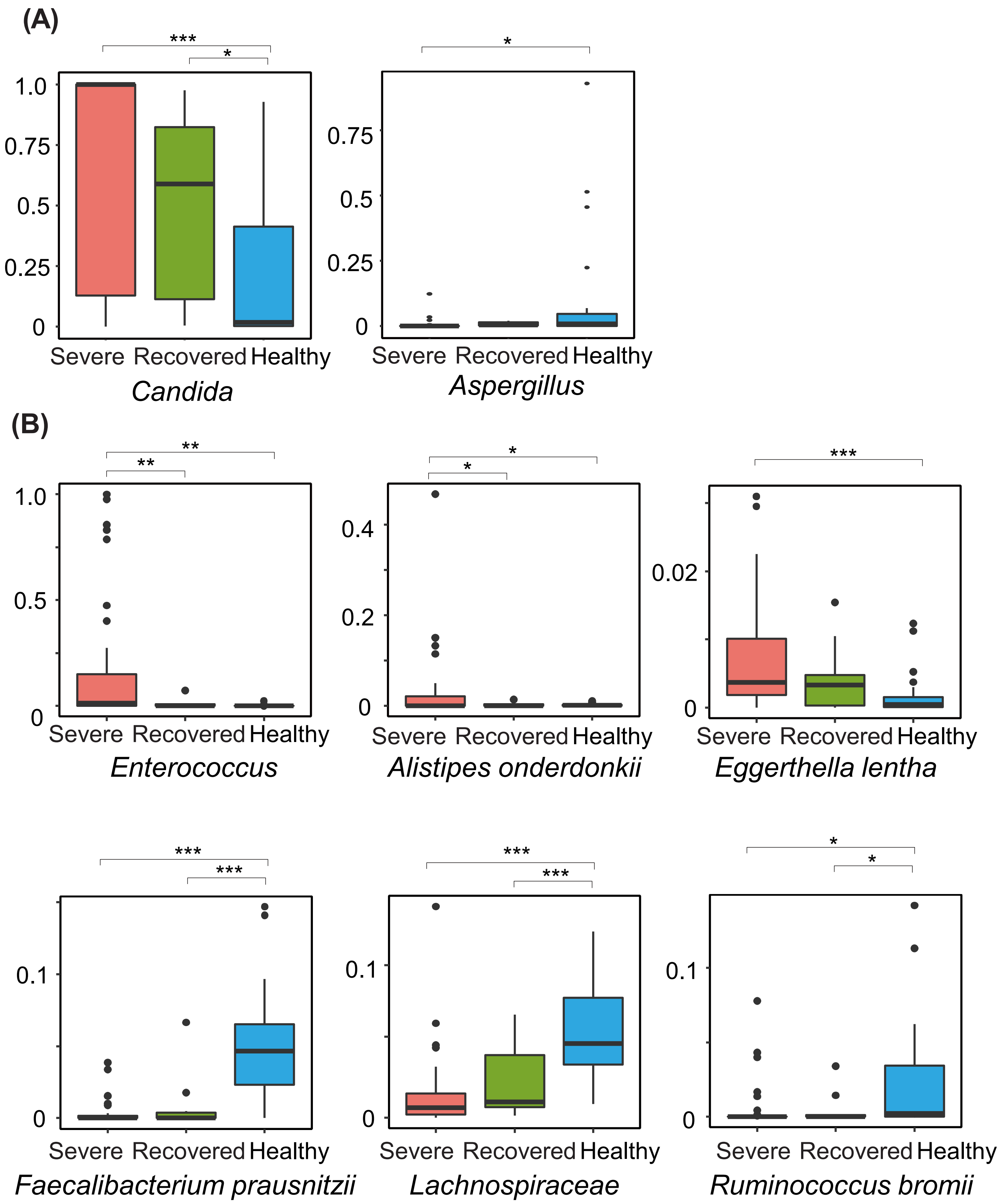
(A)
***
*
1.0
0.75
0.5
0.25
0
Severe Recovered Healthy
Candida
*
0.75
0.5
0.25
0
Severe Recovered Healthy
Aspergillus
(B)
**
**
1.0
0.5
0
Severe Recovered Healthy
Enterococcus
*
*
0.4
0.2
0
Severe Recovered Healthy
Alistipes onderdonkii
***
0.02
0
Severe Recovered Healthy
Eggerthella lentha
***
***
0.1
0
Severe Recovered Healthy
Faecalibacterium prausnitzii
***
***
0.1
0
Severe Recovered Healthy
Lachnospiraceae
*
*
0.1
0
Severe Recovered Healthy
Ruminococcus bromii
