# Supplementary material for: Longitudinal alterations of the gut mycobiota and microbiota on COVID-19 severity
Source: BMC Infect Dis. 2022 Jun 24;22:572. doi: 10.1186/s12879-022-07358-7 (PMC9233337; doi:10.1186/s12879-022-07358-7)
Supplement: Supplementary file 2 — Additional file 2: Table S1. Accession ID of SARS-CoV-2 viral sequences. [file 12879_2022_7358_MOESM2_ESM.docx]

Table S1 Accession ID of SARS-CoV-2 viral sequences

| Patient ID | Sample Type | Accession ID (GISAID) |
| --- | --- | --- |
| Cov1 | Stool | EPI_ISL_10793230 |
| Cov2 | Stool | EPI_ISL_10793231 |
| Cov3 | Stool | EPI_ISL_10793232 |
| Cov6 | Stool | EPI_ISL_10793233 |
| Cov7 | Stool | EPI_ISL_10793234 |
| Cov9 | Stool | EPI_ISL_10793235 |
| Cov13 | Stool | EPI_ISL_10793236 |
| Cov17 | Stool | EPI_ISL_10793237 |
| Cov17 | Swab | EPI_ISL_10793238 |
| Cov17 | Sputum | EPI_ISL_10793239 |
| Cov27 | Swab | EPI_ISL_10793240 |
| Cov27 | Sputum | EPI_ISL_10793241 |
| Cov32 | Stool | EPI_ISL_10793242 |
| Cov32 | Swab | EPI_ISL_10793243 |
| Cov32 | Sputum | EPI_ISL_10793244 |
| Cov38 | Stool | EPI_ISL_10793245 |
| Cov38 | Swab | EPI_ISL_10793246 |
| Cov38 | Sputum | EPI_ISL_10793247 |
| Cov40 | Stool | EPI_ISL_10793248 |
| Cov40 | Swab | EPI_ISL_10793249 |
| Cov40 | Sputum | EPI_ISL_10793250 |
